# Fully immunized child: coverage, timing and sequencing of routine immunization in an urban poor settlement in Nairobi, Kenya

**DOI:** 10.1186/s41182-016-0013-x

**Published:** 2016-05-16

**Authors:** Martin Kavao Mutua, Elizabeth Kimani-Murage, Nicholas Ngomi, Henrik Ravn, Peter Mwaniki, Elizabeth Echoka

**Affiliations:** African Population and Health Research Center, Manga Close, Nairobi, Kenya; Research Center for Vitamins and Vaccines, 5 Artillerivej, Copenhagen, Denmark; Centre for Public Health Research, Kenya Medical Research Institute, Nairobi, Kenya; Jomo Kenyatta University of Agriculture and Technology, Nairobi, Kenya; Bandim Health Project, Statens Serum Institut, 5 Artillerivej, Copenhagen, Denmark; OPEN, University of Southern Denmark/Odense University Hospital, Odense, Denmark; International Health Institute, Brown University, Providence, RI USA

**Keywords:** Fully immunized child, Coverage, Vaccination delay, Vaccination sequence

## Abstract

**Background:**

More efforts have been put in place to increase full immunization coverage rates in the last decade. Little is known about the levels and consequences of delaying or vaccinating children in different schedules. Vaccine effectiveness depends on the timing of its administration, and it is not optimal if given early, delayed or not given as recommended. Evidence of non-specific effects of vaccines is well documented and could be linked to timing and sequencing of immunization. This paper documents the levels of coverage, timing and sequencing of routine childhood vaccines.

**Methods:**

The study was conducted between 2007 and 2014 in two informal urban settlements in Nairobi. A total of 3856 children, aged 12–23 months and having a vaccination card seen were included in analysis. Vaccination dates recorded from the cards seen were used to define full immunization coverage, timeliness and sequencing. Proportions, medians and Kaplan-Meier curves were used to assess and describe the levels of full immunization coverage, vaccination delays and sequencing.

**Results:**

The findings indicate that 67 % of the children were fully immunized by 12 months of age. Missing measles and third doses of polio and pentavalent vaccine were the main reason for not being fully immunized. Delays were highest for third doses of polio and pentavalent and measles. About 22 % of fully immunized children had vaccines in an out-of-sequence manner with 18 % not receiving pentavalent together with polio vaccine as recommended.

**Conclusions:**

Results show higher levels of missed opportunities and low coverage of routine childhood vaccinations given at later ages. New strategies are needed to enable health care providers and parents/guardians to work together to increase the levels of completion of all required vaccinations. In particular, more focus is needed on vaccines given in multiple doses (polio, pentavalent and pneumococcal conjugate vaccines).

**Electronic supplementary material:**

The online version of this article (doi:10.1186/s41182-016-0013-x) contains supplementary material, which is available to authorized users.

## Background

Inadequate immunization is recognized as a major public health concern as it accounted for about 17 % of all deaths globally in children under five in 2008, preventable with immunization [1]. Achieving universal vaccination coverage for all is one of the global sustainable development targets aimed at reducing childhood mortality from preventable deaths [2]. Full vaccination coverage has been the cornerstone of immunization programmes in many countries, and it is estimated to avert an estimated two to three million deaths every year in all age groups from diphtheria, tetanus, pertussis and measles [3]. Immunization programmes have been very successful in protecting children against specific infections. Poliomyelitis infections are on the verge of complete eradication with infection cases being reported in four countries: Afghanistan, Pakistan, Nigeria and Somalia [4].

Basic immunization covers all vaccines given at any time within the first year of life and has been the focal point in evaluating immunization programmes [1, 3]. According to the World Health Organization (WHO) guidelines [5], a child is fully immunized with all basic vaccinations if the child has received Bacillus Calmette-Guerin (BCG) vaccine against tuberculosis at birth; three doses each of polio and pentavalent (diphtheria-tetanus-pertussis-hepatitis B (Hep), Haemophilus influenza type B (Hib)) vaccines at 6, 10 and 14 weeks of age; and a vaccination against measles at 9 months of age. Pneumococcal conjugate vaccine (PCV) given in three doses (6, 10 and 14 weeks) was introduced in Kenya in February 2011 and included in the routine immunization schedule [6].

Globally, full immunization coverage for children aged 12–23 months increased to 83 % [1, 7] in 2011. In Kenya, full immunization coverage for children aged 12–23 months currently stands at 79 and 75 %, respectively, when PCV is considered [8]. Only 2 % of the children aged 12–23 months had not received any vaccines [8] in 2014. However, it is also important to note that this figure can hide the variability in vaccine coverage [1, 8, 9] within Kenya and especially in urban informal settlements where the full vaccination coverage was only 44 % [10] in 2002, 58 % [11] in 2010 and 68.5 % in 2012 [12] compared to coverage in Nairobi of 73 % [13] in 2009 and 79 % in 2013 [8]. Full immunization coverage in Nairobi informal settlements depended on background characteristics of the child, mother and the household [8, 10, 12, 14]. Given the inadequate health care services in Nairobi informal settlements [15, 16] where 60–70 % of the Nairobi population resides [10, 12], identification of areas with low immunization coverage and achieving high immunization coverage in these areas become paramount for health intervention [17, 18].

The timing of vaccine is important for effectiveness and safety of the vaccine. Timely administration of vaccines has implications for the success of childhood immunization programmes, and a timely start of immunization is important in the first year of life as the transplacental immunity declines rapidly [19]. In practice, although a few children might be vaccinated early, many will be vaccinated late [20] which reduces the impact of vaccine programmes on disease burden especially in high-risk groups [21]. Vaccines given before 6 weeks of age (excluding BCG and polio at birth) have shown poor response and in some cases could be detrimental to infants as they reduce the immune response of subsequent doses [22]. Similarly, there is need to observe the minimum recommended age for different vaccines which are normally based on the youngest age group at risk for the specific infections where vaccine safety and efficacy have been demonstrated. Therefore, giving doses earlier than scheduled or given closer to each other may lead to a less optimal immune response [22]. On the other hand, when a child’s vaccine is delayed, the interval between doses/vaccines is increased and the optimal vaccine protection may not be attained [22]. Simultaneous vaccination (pentavalent, polio and PCV doses) increases the chance that a child will be fully vaccinated on time and hence improving age-specific vaccine coverage [23, 24]. Therefore, the out-of-sequencing, early or delays of vaccines may affect child survival. Several studies have documented various reasons causing delays in the administration of vaccines and their impact. A recent paper from a study in Nairobi informal settlements reveals that low birth weight infants receive BCG immunization later than normal birth weight infants [25]. Measles and BCG vaccines are known to have beneficial *non-specific effects* (NSE) when given on time while the DPT containing vaccines does not seem to [26–31].

Few studies have documented timeliness and sequencing of routine vaccinations in sub-Saharan Africa. In a study done in Ghana in 2010, 44 % of children aged 12–23 months had their measles vaccine delayed [32]. In Burkina Faso, approximately 40 % of children aged 12–23 months had their polio and pentavalent doses delayed [33]. In an earlier study in the same study area as the current study, delays in measles vaccine (MV) were estimated at 20 % among boys and 24 % among girls [34]. Immunization timeliness have been documented in several studies [25, 34–38], but almost none have looked at the levels of out-of-sequence. This gap forms the basis of this paper, which documents the levels of full immunization coverage both overall and by different factors of interest. The study also aims to document the levels of early and delayed immunization. Overall levels of out-of-sequencing of the routine vaccinations in two informal settlements in Nairobi, Kenya, are documented as well as by different factors of interest. A key strength of this study over others is the use of a longitudinal study design particularly to study timing and out-of-sequencing of routine vaccinations in urban poor settings.

## Methods

### Study setting

The study was carried out in two informal settlements of Nairobi (Viwandani and Korogocho) between 2007 and 2014 in the Nairobi Urban Health and Demographic Surveillance System (NUHDSS) ran by the African Population and Health Research Center (APHRC). The NUHDSS has been in operation since 2002 and had about 81,129 registered inhabitants in approximately 31,977 households as of December 2012. The two informal settlements are densely populated with high unemployment, crime, poor sanitation and poorer health indicators generally as compared to the whole of Nairobi. There are notable differences between the two settlements: Korogocho is more stable with less disparity in terms of gender and age distribution as compared to Viwandani which borders an industrial area and attracts migrant workers with relatively high education levels. The two communities are mainly served by private health facilities and two public health facilities located outside the area. Details of the study areas and operations of the NUHDSS have been published elsewhere [15, 16, 39].

### Study population

**Fig. 1:**
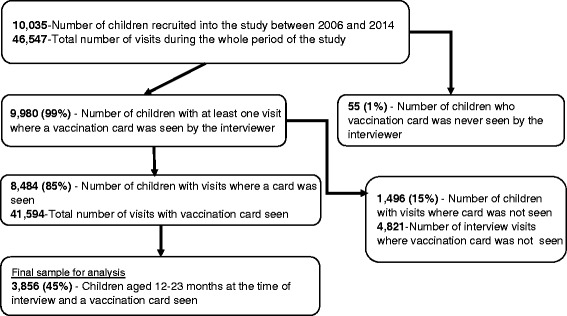
Derivation of the sample of children included in the study

This study used data from a longitudinal maternal and child health project implemented in Korogocho and Viwandani whose details have been published elsewhere [25]. The study included all children born in the study area from September 2006 to December 2013. For purpose of this study, we used data for children aged 12–23 months. All children without a vaccination card were excluded from the analysis. Figure 1 gives a diagrammatic description of how the final sample was derived.

### Study design

The study involved a longitudinal observational study design to study the outcomes. The mother-child pairs in the study were followed up every 4 months collecting information on the immunization status of the child at each visit using structured questionnaires administered by trained research assistants. We use vaccination data collected from the time of recruitment for children aged 12–23 months.

### Variables

This study assessed the levels and patterns of the routine vaccination uptake in the study area. The primary outcome variable of interest was fully immunized children (FIC) coverage. The secondary outcomes of interest were vaccination sequencing and timing of immunization (early or delayed). FIC was defined as a child who has received all the recommended basic vaccines by 12 months of age, i.e. BCG at birth, polio doses at 6 (42), 10 (70) and 14 (98) weeks (days) of age; pentavalent doses at 6 (42), 10 (70) and 14 (98) weeks (days) of age; and measles dose at 9 (274) months (days) of age. Early vaccination was defined as any vaccine given more than 4 days before recommended age for each vaccine/dose [40, 41]. In addition, for the measles vaccine, we assessed doses given more than 2 weeks before the recommended age. A vaccine is delayed if it is given more than 2 weeks after the recommended age for BCG, polio, pentavalent and PCV doses and more than a month for measles [42, 43]. Out-of-sequence (OS) was defined as either receiving (i) BCG after or with any of the other routine vaccines, (ii) any pentavalent vaccine dose with or after the MV or (iii) receiving respective pentavalent and polio doses at different days [44, 45]. A fully immunized child in out-of-sequence (FIC-OS) was defined as a child who is FIC and had at least one vaccine given in out-of-sequence.

Maternal education (none, primary and secondary or higher), ethnicity (Kikuyu, Luhya, Luo, Kamba or other), sex of child and delivery place (health facility or not), wealth status calculated using principal component analysis (lower, middle or upper) and study location (Korogocho or Viwandani) were included in the analysis. FIC coverage was also assessed by year of visit with coverage in a given year reflecting vaccines given between 1 and 2 years earlier.

### Data analysis

All children aged 12–23 months of age and a vaccination card seen were included in the analysis. For children with more than one interview visit, the earliest visit was picked for the analysis. Proportions were used to assess the levels of FIC coverage, out-of-sequence, early and delayed vaccination. The Kaplan-Meier method was used to calculate vaccination coverage curves, and the log rank test was used to compare vaccination coverage curves by FIC status. Median age at vaccination was used to assess the levels of vaccination delays. Chi-square test of independence was used to test independence between FIC coverage and levels of out-of-sequence by the different background characteristics while median test was used to test equality of medians. All tests were conducted at a 5 % level of significance. Stata software version 13.1 was used for all data management and analysis.

### Ethics, consent and permissions

The study was approved by the Kenya Medical Research Institute (KEMRI) ethical review committee. The research assistants were trained on research ethics and obtained both written and verbal informed consent from all the study respondents. The NUHDSS, on which the study was nested, also received ethical approval.

## Results

### Descriptive

The descriptive results are summarized in Table 1. A total of 3856 out of 10,035 (38 %) children met the inclusion criteria and were included in the study. Sixty-two percent of the children were excluded due to lack of an interview visit where a vaccination card was seen between 12 and 23 months of age. The sample had slightly more males (50.5 %) compared to females (49.5 %). Most of the children were delivered in a health facility (83.4 %), with majority of the mothers aged below 25 years (56.2 %) and having primary level of education (70 %). Majority of the mother-child pairs attended postnatal care (92.4 %). The last column in Table 1 summarizes FIC coverage by different background factors for children aged 12–23 months. There was no significant difference in FIC coverage between a male and female child. Significantly higher FIC coverage was observed among mothers from Viwandani area (74.1 %) compared to Korogocho (58.1 %) and among mothers who attended postnatal care (67.7 %) compared to mothers who did not attend postnatal care (53.3 %). FIC coverage varied significantly by maternal education level, parity and household wealth status.

**Table 1:** Sample characteristics and FIC coverage at 12 months of age among children aged 12–23 months

		No.	Percent	% FIC	Overall *P* value
Childs gender	Male	1925	50.5	66.0	0.391
	Female	1889	49.5	67.3	
Postnatal care	No	289	7.6	*53.3*	<0.001
	Yes	3511	92.4	*67.7*	
Mother’s age group	11–20	907	24.6	64.5	0.254
	21–24	1166	31.6	67.9	
	25–29	928	25.2	68.4	
	30–55	688	18.7	66.1	
Mother’s education level	<Primary	94	2.6	*57.4*	<0.001
	Primary	2539	70	*64.9*	
	Secondary+	995	27.4	*72.7*	
Place of delivery	Health facility	3172	83.4	*67.5*	0.017
	Not health facility	630	16.6	*62.5*	
Parity	One	1231	32.4	*71.9*	<0.001
	Two	1171	30.8	*66.4*	
	Three and above	1399	36.8	*62.2*	
Ethnicity	Kikuyu	952	25.8	*70.6*	<0.001
	Luhya	696	18.9	*62.9*	
	Luo	603	16.3	*58.4*	
	Kamba	829	22.5	*73.9*	
	Other	611	16.6	*63.5*	
Wealth status	Lower	1224	33.4	*61.1*	<0.001
	Middle	1216	33.2	*70.2*	
	Upper	1221	33.4	*69.6*	
Study site	Korogocho	1779	46.6	*58.1*	<0.001
	Viwandani	2035	53.4	*74.1*	
Year of visit	2008	921	24.1	*65.6*	<0.001
	2009	303	7.9	*68.6*	
	2010	413	10.8	*72.4*	
	2011	603	15.8	*69.5*	
	2012	941	24.7	*67.7*	
	2013	440	11.5	*60.7*	
	2014	193	5.1	*55.4*	
N		3814			

Proportions significantly different at 5 % level of significance are highlighted in italics

### Vaccination coverage

Table 2 summarizes coverage for each antigen and overall FIC coverage by year of visit. Overall FIC coverage was estimated at 66.6 % in the study area. FIC coverage was estimated at 66, 69, 72, 70, 68, 61 and 55 % for the years 2008, 2009, 2010, 2011, 2012, 2013 and 2014, respectively. BCG, oral polio vaccine (OPV) 1 and 2 and pentavalent 1 and 2 coverage were estimated at 97.1, 99.1, 96.6, 99 and 96.6 %, respectively. Lower coverages were observed for OPV 3 (82–88 %), pentavalent 3 (85–89 %) and measles (68–87 %). The coverage of PCV doses were low in 2011, the year PCV was rolled out in Kenya. Vaccination coverage of each antigen by different background characteristics are given in Additional file 1: Table S1.

**Table 2:** Immunization coverage at 12 months of age among children aged 12–23 months by year of visit

	Year of visit							
	2008	2009	2010	2011	2012	2013	2014	2008–2014
	% (#)	% (#)	% (#)	% (#)	% (#)	% (#)	% (#)	% (#)
BCG	98.4 (906)	97.7 (296)	96.9 (400)	96.8 (584)	96.3 (906)	96.6 (425)	97.4 (188)	97.1 (3705)
OPV 0	77.0 (709)	83.8 (254)	83.5 (345)	81.1 (489)	84.6 (796)	81.4 (358)	25.9 (050)	78.7 (3001)
OPV 1	99.3 (915)	99.0 (300)	100 (413)	98.3 (593)	99.0 (932)	99.5 (438)	96.9 (187)	99.1 (3778)
OPV 2	97.4 (897)	97.0 (294)	97.1 (401)	95.0 (573)	96.5 (908)	96.8 (426)	95.9 (185)	96.6 (3684)
OPV 3	82.5 (760)	86.1 (261)	87.9 (363)	84.1 (507)	88.1 (829)	88.4 (389)	84.5 (163)	85.8 (3272)
Penta 1	99.3 (915)	98.7 (299)	100 (413)	98.7 (595)	99.0 (932)	99.3 (437)	96.4 (186)	99.0 (3777)
Penta 2	97.1 (894)	96.7 (293)	97.6 (403)	96.5 (582)	96.2 (905)	96.1 (423)	94.8 (183)	96.6 (3683)
Penta 3	86.6 (798)	87.5 (265)	88.9 (367)	88.6 (534)	86.0 (809)	88.2 (388)	85.5 (165)	87.2 (3326)
PCV 1				61.4 (248)	90.6 (853)	97.7 (430)	93.3 (180)	86.5 (1711)
PCV 2				45.3 (183)	83.2 (783)	94.1 (414)	91.2 (176)	78.7 (1556)
PCV 3				31.4 (127)	70.1 (660)	80.0 (352)	79.3 (153)	65.3 (1292)
Measles	81.2 (748)	82.8 (251)	86.7 (358)	85.7 (517)	81.6 (768)	73.0 (321)	68.4 (132)	81.1 (3095)
FIC 8	65.6 (604)	68.6 (208)	72.4 (299)	69.5 (419)	67.7 (637)	60.7 (267)	55.4 (107)	66.6 (2541)
N	921	303	413	603	941	440	193	3814

### Median age of vaccination

The median age and interquartile range (IQR) of each antigen are summarized for non-FIC children (Additional file 2: Table S2a) and FIC children (Additional file 2: Table S2b) by background characteristics. The median age for BCG was estimated at 6 days (IQR 1–14) for FIC and 8 days (IQR 2–17) for non-FIC children. Median age for BCG varied significantly by postnatal care attendance, delivery place, parity and ethnicity among FIC children and delivery place and ethnicity among non-FIC children. Median age for the third dose of pentavalent was estimated at 107 (102–114) and 110 (103–126) days for non-FIC and FIC children, respectively. The median age for the third dose of polio was estimated at 111 (104–129) days for non-FIC and 107 (102–116) days for FIC children with significant differences observed by maternal level of education, delivery place, parity, ethnicity, wealth status and location for both FIC and non-FIC children. Median age for the MV was estimated at 282 (275–294) days among FIC children and 290 (277–323) days for non-FIC children with significant differences observed by child’s gender for FIC children and by postnatal care, mother’s age, parity, ethnicity, wealth status and location for non-FIC children.

**Fig. 2:**
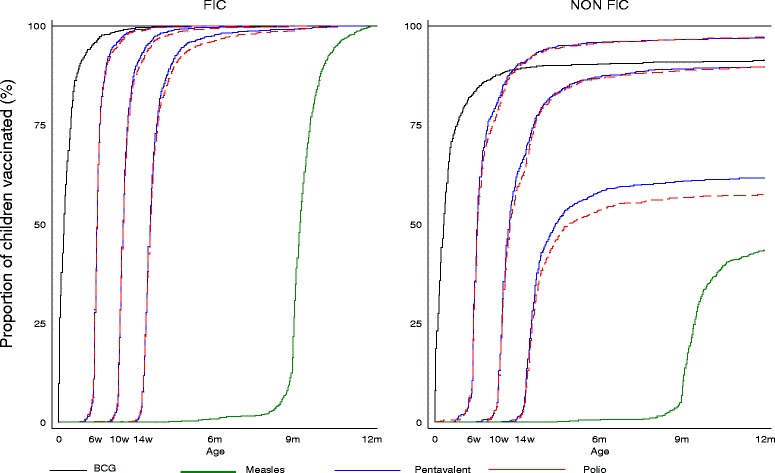
Vaccine coverage curves by 12 months among children aged 12–23 months

The coverage curves are shown in Fig. 2 for the whole period and for each year’s visit in the additional file (Additional file 3: Figure S1a and S1b). The curves for each vaccine by definition end at 100 % for FIC children. Vaccination timing among FIC are remarkable especially for the polio and pentavalent doses which are almost upright apart from a few children who received them a bit later. The MV and BCG coverage curves appear less upright. The FIC coverage curves for the OPV doses improved over the years. The coverage curves among the non-FIC children do not reach 100 % and are less upright compared to FIC children which shows that more non-FIC children have their vaccines delayed compared to FIC. Results from log rank tests showed significant differences in Kaplan-Meier curves between third doses of polio and pentavalent (*P* value 0.004) and non-significant differences between the first doses (*P* values = 0.242) and second doses (*P* value = 0.054) of polio and pentavalent vaccines in all children, respectively. Significant differences (*P* value <0.001) in Kaplan-Meier curves of each antigen by FIC status were observed from log rank tests. Additional file 4: Table S3 shows children are receiving their vaccines earlier than recommended ages: more than 4 days for OPV 1 (3.3 %) and pentavalent 1 (2.7 %) and MV (8.4 %). Five percent of the children received MV more than 14 days before the appropriate age. The proportion of early immunization is high among the non-FIC compared to FIC children for pentavalent doses (*P* values, 0.005, 0.029 and 0.001 for first, second and third doses, respectively) and the first polio dose (*P* value = 0.010).

### Vaccine sequence

Table 3 summarizes levels of FIC-OS. Overall, 21.7 % of FIC children were FIC-OS. The levels of FIC-OS were significantly higher among mothers not attending postnatal care (29.2 %), those that did not deliver at a health facility (31.0 %) and those from the Korogocho settlement area (30.5 %) and also differed significantly by ethnicity group and household wealth status. The main cause of being FIC-OS was not receiving pentavalent and polio doses together (18.2 %).

**Table 3:** Proportion of children receiving vaccination in out-of-sequence (FIC-OS) by 12 months of age among children aged 12–23 months by different background factors

	Late BCG (%)	Pentas<>OPVs (%)	Penta>=MV (%)	FIC-OS (%)	N	Overall *P* value
Overall	4.0	18.2	0.8	21.7	2541	–
Sex						
Male	4.5	17.5	0.9	21.3	1270	0.646
Female	3.5	18.9	0.8	22.1	1271	
Postnatal						
No PN	6.5	*24.0*	0.6	*29.2*	154	0.021
PN	3.9	*17.7*	0.8	*21.2*	2378	
Mother’s age group						
11–20	4.3	20.2	0.9	24.1	585	0.185
21–24	4.3	16.5	0.8	20.3	792	
25–29	3.0	17.5	0.8	20.0	635	
30–55	4.0	19.3	0.7	22.6	455	
Education						
<Primary	1.9	*25.9*	0.0	25.9	54	0.080
Primary	4.0	*18.7*	0.9	22.2	1647	
Secondary+	3.6	*15.2*	0.4	18.5	723	
Delivery place						
Health facility	*2.5*	17.7	*0.6*	*20.0*	2140	
Non-HF	*12.2*	20.3	*2.0*	*31.0*	394	<0.001
Parity						
Parity1	4.1	18.0	*0.2*	21.5	885	0.543
Parity2	3.9	17.2	*1.2*	20.6	778	
Parity3+	4.1	19.1	*1.1*	22.9	870	
Ethnicity						
Kikuyu	2.7	*20.1*	*0.0*	*22.2*	672	0.001
Luhya	5.0	*20.3*	*1.4*	*25.1*	438	
Luo	5.4	*22.2*	*1.4*	*26.7*	352	
Kamba	3.8	*14.4*	*0.7*	*17.9*	613	
Other	3.6	*14.7*	*1.0*	*17.5*	388	
Wealth status						
Lower	4.3	*25.4*	1.2	*29.0*	748	<0.001
Middle	3.5	*17.7*	0.5	*20.5*	854	
Upper	4.2	*12.6*	0.8	*16.7*	850	
Study site						
Korogocho	4.4	*26.9*	1.1	*30.5*	1033	
Viwandani	3.8	*12.2*	0.7	*15.7*	1508	<0.001

Proportions significantly different at 5 % level of significance are highlighted in italics

*Late BCG* BCG given together or after any pentavalent or measles, *Pentas<>OPVs* corresponding pentavalent and polio not given together, *Penta>=MV* pentavalent given together or after measles, *FIC-OS* fully immunized child by 12 months with either late BCG, Pentas<>OPVs or Penta>=MV

Table 4 summarizes reasons for a child not being FIC in the study area. Approximately 60 and 17 % of children who were not FIC were missing only one or two vaccines, respectively. The main vaccines missing were OPV 3 (42 %), MV (47 %) and pentavalent 3 (38 %). The above-mentioned reasons are consistently observed in all years where data was available (see Additional file 2: Figure S1a and S1b)

**Table 4:** Proportion of non-FIC children with missing vaccines by number and type of vaccines among children aged 12–23 months

	2008	2009	2010	2011	2012	2013	2014	Total
	%	%	%	%	%	%	%	%
Number of vaccine missing								
0 (FIC after 12 months)	6.9	6.3	6.1	7.6	7.2	3.5	5.8	6.4
1	58.4	62.1	59.6	55.4	56.9	71.1	70.9	60.6
2	18.0	16.8	19.3	18.5	19.7	11.6	9.3	17.0
3	9.8	4.2	7.9	8.7	6.9	4.6	4.7	7.3
*4*	4.1	4.2	3.5	3.8	4.3	5.2	2.3	4.1
*5*	1.9	4.2	2.6	3.3	3.3	2.9	2.3	2.8
*6*	0.6	0.0	0.9	0.5	0.7	0.0	0.0	0.5
*7*	0.3	2.1	0.0	2.2	0.7	0.6	4.7	1.1
*8*	0.0	0.0	0.0	0.0	0.3	0.6	0.0	0.2
Vaccine missing by type								
BCG	4.4	6.3	11.4	9.2	10.9	8.7	2.3	7.9
OPV 1	1.6	2.1	0.0	4.3	1.6	1.2	4.7	2.0
OPV 2	7.3	8.4	10.5	15.2	9.5	8.1	5.8	9.3
OPV 3	50.5	43.2	43.9	51.1	35.5	29.5	32.6	41.8
Penta 1	1.9	4.2	0.0	3.8	3.0	1.7	8.1	2.8
Penta 2	8.5	10.5	8.8	10.9	11.8	9.8	11.6	10.2
Penta 3	38.8	40.0	40.4	37.0	43.4	30.1	32.6	38.3
MV	42.6	46.3	39.5	37.0	46.1	63.0	59.3	46.5
N	317	95	114	184	304	173	86	1273

## Discussion

This study looks at the levels of coverage of fully immunized children (FIC) by 12 months, timing of vaccinations (early and delays) and sequencing of the routine childhood vaccination in children aged 12–23 months in urban informal settlement in Nairobi, Kenya. FIC coverage was estimated at 66.6 %. Individual antigen coverage were above 90 % apart from MV, OPV 3 and pentavalent 3. Overall FIC coverage was shown to depend on postnatal care attendance, education, parity, ethnicity, household wealth status and location. The median age of the different vaccines revealed significant delay in immunization among the non-FIC children compared to FIC children. Proportion of fully immunized children in out-of-sequence (FIC-OS) was estimated at 22 %. FIC-OS differed significantly by postnatal care attendance, delivery place, ethnicity, household wealth status and location. The study highlights main reasons for not being FIC and identifies levels and sources of FIC-OS.

The overall FIC coverage in this study was higher compared to 44 % in a previous study conducted in approximately all informal settlements in Nairobi [10] in 2002. The estimates are similar to (68 %) results obtained from a cross-sectional study targeting approximately all informal settlements in Nairobi [14] conducted in 2012. The FIC coverage in this study still lags behind Nairobi (81 %) and national estimates (79 %) [46]. The increase in FIC coverage (57–68 %) in the study area may be attributed to efforts made by the ministry of health and other stakeholders to improve the uptake of health services and awareness from interventions conducted in the study area, even though more needs to be done to reduce the gap existing between informal settlements and other parts of Nairobi. Increases in immunization coverage have been reported in other low- and middle-income countries over the years [7, 47]. Our results show FIC coverage differed by different background characteristics. FIC coverage was high among mothers who attended postnatal care, and this is expected as their children have more chances of getting the vaccines than those who do not make any follow-up contact with a health centre after delivery. FIC coverage was higher among children of mothers with high education level, resonating with other studies [11, 13, 14, 48]. Mothers with lower parity had higher coverage compared to mothers with higher parity, which has been found in other studies [11, 49]. The more children a mother has the more constraints on the little resources available especially in informal settlements where levels of poverty are high and affect health care utilization [20]. Ethnicity also played a role in determining FIC coverage. FIC coverage was higher among Kamba and Kikuyu ethnic groups compared to other ethnic groups. Similar results were found in other studies done in Kenya, and this has been linked to cultural differences in addition to education and income disparities among the different ethnicities [11, 14, 34]. FIC coverage was higher among children from households with higher wealth status. This has been documented in other studies done in Kenya [8, 11, 13, 14] and India [48] where health outcomes are better off among the wealthier in the community compared to the less wealthy households. FIC coverage was found to be higher in Viwandani compared to Korogocho study area in line with earlier studies [11]. Viwandani area is next to an industrial area, and residents here are better off than Korogocho residents.

This study showed higher coverage for vaccines given during an infant’s early part of life and lower for vaccines given later in life specifically OPV 3, pentavalent 3, PCV 3 and MV. This resonates well with previous findings in the study area [11, 13] and other studies conducted in Burkina Faso, Nigeria and South Africa [33, 36, 37]. The issue of not completing recommended doses of a vaccine is a concern. A child is protected optimally from specific infections if the child received all the three doses. When a dose is skipped, delayed or missed altogether, the child becomes vulnerable from the specific infection and also ‘herd’ immunity is compromised [50]. The low coverage of MV poses similar concerns. Studies have shown MV and BCG vaccines to be having non-specific beneficial effects on child survival [26–28, 31, 51–58]. The high number of infants missing out on MV vaccines could be missing out on these benefits.

The study showed an initial increase of FIC coverage between 2008 and 2010 followed by a decrease between 2011 and 2014. Further investigation is needed in the study area to understand this scenario. In general, there has been an increase in FIC coverage from the study area compared to similar studies conducted in the current area [10, 11]. Another cross-sectional study conducted in approximately all informal settlements in Nairobi in 2012 showed equivalent FIC coverage [14]. Though this study provides evidence of increase in FIC coverage over the last decade [10], the coverage is still lower in poor urban settlements as compared to estimates from other urban areas and Nairobi in particular [8, 13]. Children missing MV, OPV 3 and pentavalent 3 vaccines were identified by the study as the main reasons of not being fully immunized. Similar observations were made by other studies conducted in other settings [36, 59].

A substantial number of children started their routine immunization much earlier than the recommended age. Similar result has been reported elsewhere in Nigeria, Mozambique and Guinea [37, 60]. Studies have shown that vaccines given more than 4 days earlier than the recommended age may not be optimally effective [24] and one may need to re-vaccinate.

The study established substantial levels of delays in BCG, OPV 3, pentavalent 3 and measles vaccine coverage. This is consistent with other studies in sub-Saharan Africa [37]. The study showed most of the specific vaccines delays were associated with postnatal attendance, ethnicity, education level and delivery place, social economic status and location of the household. These same factors were identified as being associated with being FIC by 12 months of age. Similar results have been found in other studies as determinants of vaccine delays [32, 33, 45, 61, 62].

This study provides evidence of children receiving vaccines in a different sequence than recommended. The main contributor of being FIC-OS was identified as not receiving the pentavalent and corresponding polio dose together. This highlights levels of missed opportunities in immunization programmes as the child had contact with a health care person and was only given one vaccine instead of two. This may be occasioned by vaccine stock-outs.

Overall, the study underscores the importance of a child being fully immunized and getting the vaccines on time and in the correct sequence. The existence of disparities even among the underprivileged in these population have implications that policymakers need to be aware of. The results highlight the simple measures which can be taken to improve on coverage, timing and sequencing of the vaccines. This means that the lower immunization coverage and age-specific vaccination coverage can easily be improved by targeting the disadvantaged groups. Special focus is needed on the uptake of all the three doses for polio, pentavalent and PCV vaccines, at the same time making sure a child is given all the doses that are supposed to be given at the same day when there is contact with a health centre. Ensuring that parents/guardian know the importance of the children receiving all the three doses of the key vaccination will improve not only coverage but also making sure the child has received the vaccine at the right time.

This study was conducted in an urban informal settlement, and therefore, the estimated coverages, levels of delays and out of sequence may only represent similar populations. A major limitation of this study was that we did not see a vaccination card for a large number (62 %) of those recruited during the visits between 12 and 23 months of age and hence they were excluded from the analysis. However, on the positive side, the exclusion eliminated the possibility of introducing recall and survival bias in the analysis. Despite the exclusion, the analysis still included a reasonable sample size. However, excluding children without an observed vaccination card may impact the internal validity of the results within the target population as vaccine coverage among these children may differ in substantial ways from children with an observed vaccination card. The major strength of the study was the longitudinal nature of the study which gives an opportunity of visiting the respondent several times. This helps in getting better estimate and trend data compared to cross-sectional studies which only give a snapshot of vaccination coverage at a given time.

## Conclusions

The study reveals high levels of missed opportunities in the administration of routine childhood vaccinations. A substantial number of children were not fully immunized by the end of their first year of life; even when they are fully immunized, a sizeable number received their vaccines inappropriately, either early, delayed or in a different sequence from the recommended schedule. New strategies are needed to enable health care providers and parents/guardians to work together to increase the levels of completion of all required vaccines. In particular, more focus is needed on measles and vaccines given in multiple doses (polio, pentavalent and pneumococcal conjugate vaccine) to make sure children receive all the three doses. This study contributes to the documentation of patterns of routine immunization uptake in urban poor settlements in Kenya and similar settings.

## Additional files

Additional file 1: Table S1. Proportion of fully immunized children by 12 months of age by different background factors. The file lists the vaccination coverage of each antigen by different background characteristics for children aged 12-23 months. (XLSX 14 kb) Additional file 2: Median age of vaccination. Table S2a: Median age of vaccination (days) among non-FIC children aged 12–23 months. Table S2b: Median age of vaccination (days) among FIC children aged 12–23 months. (XLSX 17 kb) Additional file 3: Vaccine coverage curves. Figure S1a: Vaccine coverage curves by 12 months among children aged 12–23 months by year of visit and FIC status (2008–2011). Figure S1b: Vaccine coverage curves by 12 months among children aged 12–23 months by year of visit and FIC status (2012–2014). (PDF 117 kb) Additional file 4: Table S3. Proportion of children aged 12–23 months given vaccines more than 4 (14 for MV) days before the recommended age. The file shows children are receiving their vaccines earlier than recommended ages. (XLSX 10 kb)

## Abbreviations

APHRC: African Population and Health Research Center
BCG: Bacillus Calmette-Guerin
FIC: fully immunized child
FIC-OS: fully immunized child in out-of-sequence
KEMRI: Kenya Medical Research Institute
MV: measles vaccine
NSE: non-specific effects
NUHDSS: Nairobi Urban Health Demographic Surveillance System
OPV: oral polio vaccine
PCV: pneumococcal conjugate vaccine
