# Supplementary material for: Fully immunized child: coverage, timing and sequencing of routine immunization in an urban poor settlement in Nairobi, Kenya
Source: Trop Med Health. 2016 May 16;44:13. doi: 10.1186/s41182-016-0013-x (PMC4940963; doi:10.1186/s41182-016-0013-x)
Supplement: Additional file 3: — Vaccine coverage curves. Figure S1a: Vaccine coverage curves by 12 months among children aged 12–23 months by year of visit and FIC status (2008–2011). Figure S1b: Vaccine coverage curves by 12 months among children aged 12–23 months by year of visit and FIC status (2012–2014). (PDF 117 kb) [file 41182_2016_13_MOESM3_ESM.pdf]

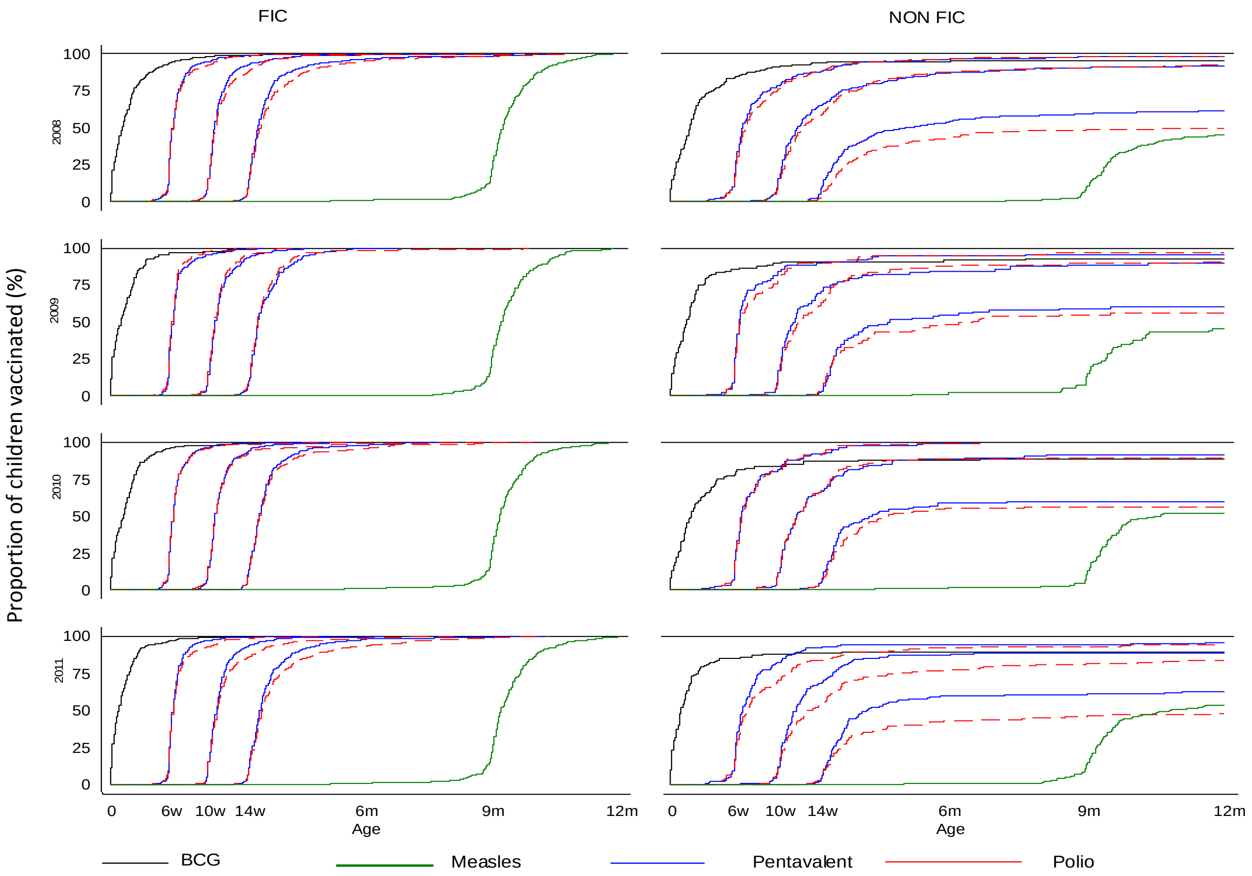

Figure A1a: Vaccine coverage curves by 12 months among children aged 12-23 months by year of visit and FIC status (2008-2011)

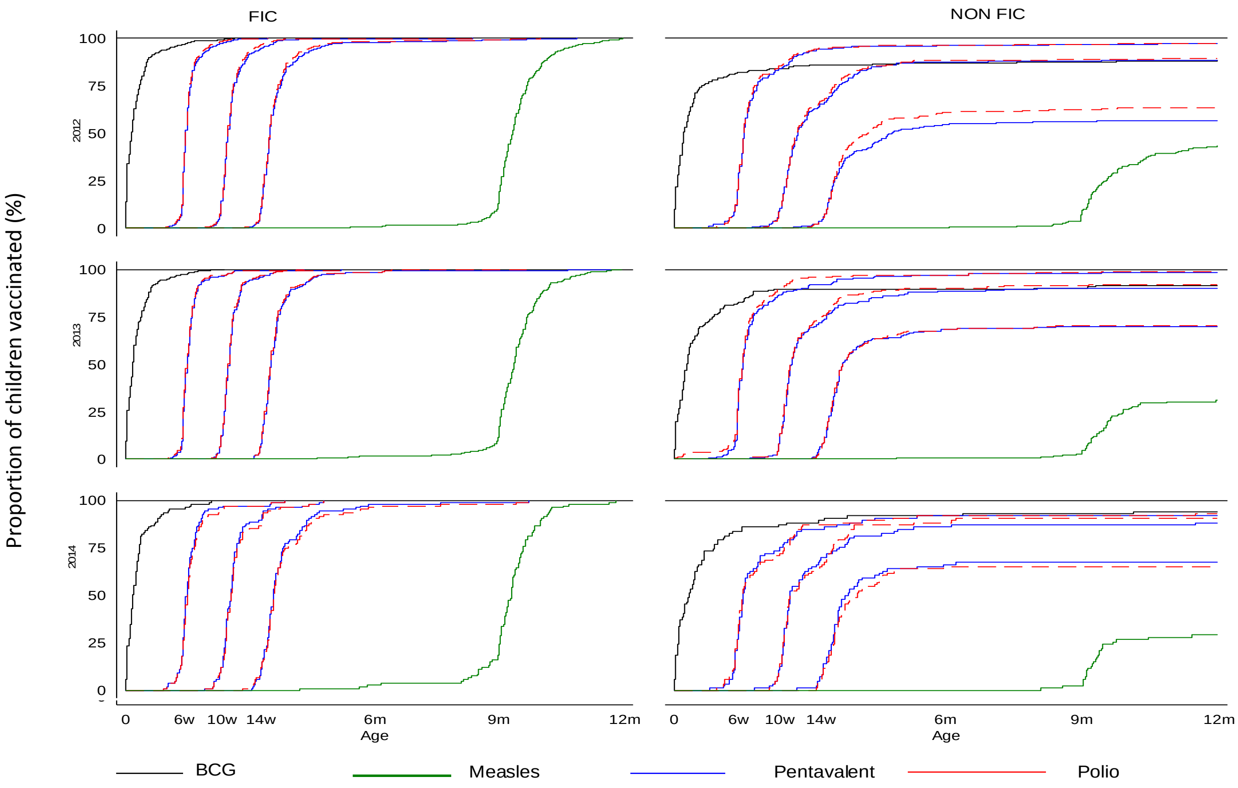

Figure A1b: Vaccine coverage curves by 12 months among children aged 12-23 months by year of visit and FIC status (2012-2014)
